# Supplementary material for: Development of Anti-LRRC15 Small Fragments for Imaging Purposes Using a Phage-Display ScFv Approach
Source: Int J Mol Sci. 2022 Oct 21;23(20):12677. doi: 10.3390/ijms232012677 (PMC9604383; doi:10.3390/ijms232012677)
Supplement: Supplementary file 1 [file ijms-23-12677-s001.zip › ijms-1927729-supplementary/SM/Supplementary data S3 ORF diversity.pdf]

>31
QVQMKQSGAEVVRPGVSVKISCKGSGYTFTDYPMHWVKQSHAKSLEWIGFISTYYGDANYNQKFKGKATMTVDKSSNTAYMEL
ARLTSEDSAIYYCARSRYGSSYVDYWGQGTTLTVSSASGGGGSGGGGSGGGGSAGEIVLTQSPVIMSASLGERVTMTCTASSS
VSSSYLHWYQQRPGSSPKLWIYSTSNLASGVPARFSGSGSGTSYSLTISSMEAEDAATYYCQQFSGYPYTFGGGTRLVLKRAA
AAHHHHHHGAAEQKLISEEDLNGAA*
>43
QVQLKESGAELMKPGASVKISCKATGYTFTSYWIDWIKQRPGHGLEWLGEILPGSGSTKYNEKFKGKATFTADTSSNIAYMQL
NSLTSEDSAVYYCARNGNYVPDYFDYWGQGTTLTVFSASGGGGSGGGGSGGGGSAGENVLTQSPAIMSASPGEKVTMTCSASS
SVSYMNWYQQKPGSSPKICIYGISNLASGVPARFSGSGSGTSYSLSLSSMEAEDAAIYYCQQWNYPLTFGAGTKLEMKRAAAA
HHHHHHGAAEQKLISEEDLNGAA*
>67
EVQLQQPGPEVVRPGVSVKISCKGSGYTFTDYAMHWVKQSHAKSLEWIGIISTYNGNTNYNQKFKGKATMTVDKSSSTAYMEL
ARLTSEDSAIYYCAREGGVRGYAMDYWGQGTSVTVFAASGGGGSGGGGSGGGGSAGDIVLTQSPASLAVSLGQRATISCRASE
SVEYYGTSLMQWYQQKPGQPPKLLIYAASNVESGVPARFSGSGSGTDFSLNIHPVEEDDIAMYFCQQSRKVPWTFGGGTKLEI
KRAAAAHHHHHHGAAEQKLISEEDLNGAA*
>79
QVQLKQSGAELVRPGVSVKISCKGSGYTFTDYGMHWVKQTHAKSLEWIGVISTYYGDSNYNQKFKGKATMTVDKSSSTAYMEL
ARLTSEDSAIYYCARSRYGSSYVDYWGQGTTLTVSAASGGGGSGGGGSGGGGSAGETTVTQSPASLSMAIREKVTIRCITSTD
IDDDMNWYQEKPGEPPKLLISEGNILRPGVPSRFSSSGYGTDFLFTIENMLSEDVADYYCLQSDNLPLTFGAGTKLDLKRAAA
AHHHHHHGAAEQKLISEEDLNGAA*
>08
EVKLVESGGGLVKPGGSLKLSCAASGFTFSSYAMSWVRQTPEKRLEWVASISSGGSTYYPDSVKGRFTISRDNARNILYLQMS
SLRSEDTAMYYCARGGRYYGSSDAMDYWGQGTSVTVSAASGGGGSGGGGSGGGGSAGEIVLTQSPAIMSASPGEKVTMTCSAS
SSINYMHWYQQKPGTSPKRWISDTSKLASGVPARFSGSGSGTSYSLTISSMEAEDAATYYCHQRSSYPWTFGGGTKLEIKRAA
AAHHHHHHGAAEQKLISEEDLNGAA
>20
QVQLKESGPGLVAPSQSLSITCTVSGFSLTSYGVHWVRQPPGKGLEWLGVIWAGGNTNYNSALMSRLSINKDNSKSQVFLKMN
SLQTDDTAMYYCARDDSEAYWGQGTLVTVSAASGGGGSGGGGSGGGGSAGEIVLTQSPAILSASPGEKVTMTCRASSGVSYMH
WYQQKPGSSPKPWIYATSNLASGVPTRFSGSGSGTSYSLTISRMEAEDAATYYCHQRSSYPWTFGGGTKLELKRAAAAHHHHH
HGAAEQKLISEEDLNGAA*
>32
QVQLKQSGAELMKPGASVKISCTAAGYTFNNYWIEWVKQRPGHGLEWIGEILPGSGSTNYNEKFKVKATFTVDTSSNTAYMQL
SSLTSEDSAVYYCARKSYHGSNYGYFDVWGAGTTVTVSAASGGGGSGGGGSGGGGSAGDVVMTQTPLSLPVSLGDQASISCRS
SQSLVHNNGNTYLHWYLQKPGQSPKLLLYKVSNRFSGVPDRFIGSGSGTDFTLKISRVEAEDLGVYYCWQGTHFPRTFGGGTK
LELKRAAAAHHHHHHGAAEQKLISEEDLNGAA*
>44
EVQLLETGGGLVQPGGSRKLSCAASGFTFSSFGMHWVRQAPDKGLEWVAYISSGSSIIYYADTVKGRFTISRDNPKNTLFLQM
TSLRSEDTAMYYCARGNYYFDYWGQGTTLTVFAASGGGGSGGGGSGGGGSAGETTVTQSPASLSVATGEKVTVRCITSTDIDG
DINWYQQKPGEPPKLLISEGNTLRPGVPSRFSSSGYGTDFVFTIENTLSEDVADYYCLQSDNMPYTFGGGTKLEIKRAAAAHH
HHHHGAAEQKLISEEDLNGAA*
>81
EVQLQQSGPEVVRPGISVKISCKGSGYTFTDYAMHWVKQSHAKSLEWIGVISTYSGNTNYNQKFKGKATMTVDKSSSTAYMEL
ARLTSEDSAIYYCARYDGYWYFDVWGAGTTVTVSSASGGGGSGGGGSGGGGSAGEIVLTQSPAILSASPGEKVTMTCRASSSV
SYIHWYQQKPGSSPKPWIYATSNLASGVPARFSGSGSGTSYSLTISRVEAEDAATYYCQQWSSNPLTFGAGTKLELKRAAAAH
HHHHHGAAEQKLISEEDLNGAA*
>10
EVQLQQPGAELVKPGASVKLSCKASGYTFTSYYMDWVKQRPGQGLEWIGEINPSNGGTNFNXKFKSKATLTVDTSSSTAYMQL
SSLTSEDSAFYYCTMGNYYAMDCWGQGTSVTVSAXSGGGGSGGGGSGGGGSAGDIVMTXAAPSVPVTPGESVSISCRSSKSLL
HXNGNTYLYWFLQRPGQSPQLLIYRMSNLASGVPDRFSGSVSGTAFTLRISRVEAEDVGVYYCAQNLELPWTFGGGTKLAIKR
AAAAHHHHHHGAAEQKLISEEDLNGAA*
>22
EVKLMESGGGLVQPGGSLKLSCAASGFTFSTYGMSWVRQTPDKRLALVASINNNGGTTYYPDSVKGRFTISRDNAKNTLYLQM
SSLKSEDTATYYCARDDGYVPPMDYWGQGTSVTVFSASGGGGSGGGGSGGGGSAGETTVTQSPASLSMAIGEKVTIRCLTSTD
IDDDMNWYQQKPGEPPKLLISDGNTLRPGVPSRFSSSGYGTDFVFTIENMLSEDVADYYCLQSDNLPYTFGGGTKLEIKRAAA
AHHHHGAAEQKLISEEDLNGAA*
>46
EVQLVESGGGLVKPGGSLKLSCAASGFAFSSYDMSWVRQTPEKRLELVAAINSNGGSTYYPDAVKGRFTISRDNAKDTLYLQM
SSLKSEDTALYYCARKRGSSPYAMDYWGQGTSVTVSSASGGGGSGGGGSGGGGSAGDIVMTQAAPSVPVTPGESVSISCRSSE
SLLHSNGHTYLYWFLQRPGQSPQLLIYRMSNLASGVPDRFSGSGSGTAFTLRISRVEAEDVGVYYCAQNLELPWTFGGGTKLE
IKRAAAAHHHHHHGAAEQKLISEEDLNGAA*
>70
QVQLKQSGPDLVKPSQSLSLTCTVTGYSFTSDYNWHWIRQFPGDKLEWMGYIHFSGSTHYNPSLKSRISITRDTSKNQFFLQL
NSVTTEDTATYFCACHFYGYFDYWGQGTTLTVFAASGGGGSGGGGSGGGGSAGETTVTQSPASLSVATGEKVTIRCITSTDID
DDMNWYQQKPGEPPKLLISEGNSLRPGVPSRFASSGYGTDFVFTIENTVSEDVADYYCLQSDNMPLTFGAGTKLVLKRAAAAH
HHHHHGAAEQKLISEEDLNGAA*
>82
QVQLQQSGPELVKPGASVKISCKGSGYTFTDYAMHWVKQTHAKSLEWIGVISTYYGDANYDQKFKDKATMTVDKSSSTAYMEL
ARLTSEDSAIYYCARSRYGSSYVDYWGQGTTLTVSSASGGGGSGGGGSGGGGSAGDVVMTQTPLSLPVSLGDQASISCRSSQS

LVHSNGNTYLHWYLQKSGQSPKLLIYKVSNRFSGVPDRFSGSGSGTDFTLKISRVEAEDLGVYFCSQSTHVPWTFGGGTKLEI
KRAAAAHHHHHHGAAEQKLISEEDLNGAA*
>94
DVQLQESGGGLVQPGGSLKLSCAASGFDFSRYWMSWVRQAPGKGLEWIGEINPDSSTINYTPSLKDKFIISRDNAKNTLYLQM
SKVRSEDTALYYCARRGTVVVPAMDYWGQGTSVTVSAASGGGGSGGGGSGGGGSAGDIVMTQAAPSVPVTPGESVSISCRSSE
SLLHSNGNTYLYWFLQRPGQSPHLLIYRMSNLASGVPDRFSGSGSGTAFTLRISRVEAEDVGVYYCMQHLEYPLTFGAGTKLV
LKRAAAAHHHHHHGAAEQKLISEEDLNGAA*
>76
MITPSFGAFFLEIFNVKYLLPTAAAGLLLLAAHPAMAEVQLQESGPGLVAPSQSLSITCTVSGFSLTSYGVHWVRQPPGKGLE
WLGIIWAGGSTNYNSALMSRLSISKDNSKSQVFLKMYSLQTDDTAMYYCARFGYPFDYWGQGTTLTVFSASGGGGSGGGGSGG
GGSAGDIQMTQSPASLSASVGETVTITCRASGNIHNYLAWYQQKQGKSPQLLVYNAKTLADGVPSRFSGSGSGTQYSLKINSL
QPEDFGTYYCQHFWITPPTFGGGTKLELKRAAAAHHHHHHGAAEQKLISEEDLNGAA
>05
QVQLQQSGPELVKPGASVKMSCKTSGYTFTDYNIHWVKQSHGKSLEWIGYIYPYNGGSGYNQKFKTKATLTVDSSSSTAYMEL
RSLTSEDSAVYYCARRGYDHMRDTMDYWGQGTSVTVSSASGGGGSGGGGSGGGGSAGEILLTQSPAIMSASPGEKVTMTCSAS
SSVSYMYWYQQKPGSSPRLLIFDTANLASGVPVRFSGSGSGTSYSLTISRMEAEDAATYYCQQWSGYPLTFGAGTKLDLKRAA
AAHHHHHHGAAEQKLISEEDLNGAA*
>17
EVQLQQSGAELVRPGTSVKVSCKASGYAFTNYLIEWIKQRPGQGLEWIGVINPGSGSSDYNEKFKAKATLTADRSSSTAYMQL
SSLTSDDSAVYYCARTARATYAMDYWGQGTSVTVSAASGGGGSGGGGSGGGGSAGEIVLTQSPAIMSASPGEKVTMTCSASSS
VTYMQWYQQKSGTSPKRWIYDTSKLASGVPARFSGSRSGTSYSLTISSMEAEDAATYYCQQYSGYPLTFGAGTKLDLKRAAAA
HHHHHHGAAEQKLISEEDLNGAA*
>29
EVKLLESGPGLVKPSQSLSLTCTVTGYSVTSDYAWNWIRQFPGNKLEWMGYISYSGTTSYNPSLKSRISITRDTSKNQFFLQL
NSVTTEDTATYYCARGDYDGFDYWGQGTTLTVSSASGGGGSGGGGSGGGGSAGEIVLTQSPAIMSASLGEEITLTCSASSSVS
YMHWYQQKSGTSPKLLIYSTSNLASGVPSRFSGSESGTFYSLTISSVEAEDAADYYCHQWSSYRTFGGGTKLAIKRAAAAHHH
HHHGAAEQKLISEEDLNGAA*
>53
EVQLEESGPGLVAPSQSLSITCTVSGFSLTSYGVHWVRQPPGKGLEWLGVIWAGGGTNYNSALMSRLSISKDNSKSQVFLNMN
GLQTDDTAMYYCARDDSEAYWGQGALVTVFSASGGGGSGGGGSGGGGSAGENVLTQSPAIMSASPGEKVTMTCSASSSVSYMH
WYQQKSGTSPKRWIYDTSKMASGVPARFSGSGSGTSYSLTISSMEAEDAATYYCQQWSSNPPTFGAGTKLDLKRAAAAHHHHH
HGAAEQKLISEEDLNGAA*
>65
QMQLKESGPGLVKPSQSLSLTCTVTGHTITSDYAWNWIRQFPGNKLEWMGYISYSGSTTYNPSLKSRISITRDTSKNQFFMQL
KSVTTEDTAKYYCADGYYYVMDHWGQGTSVTVSAASGGGGSGGGGSGGGGSAGQIVLTQSPAIMSASPGEKVTITCSASSSVS
YMHWFQQKPGTSPKLWIYSTSNLASGVPARFSGSGSGTSYSLTISSMEAEDAATYYCHQRSSYPWTFGGGTKLDIKRAAAAHH
HHHHGAAEQKLISEEDLNGAA*
>77
EVQLLETGGGLVKPGGSLKLSCAASGFIFSDYYMYWVRQTPEKRLEWVATISDGGSYTYYPDSVKGRFTISRDNAKNTLHLQM
SSLRSEDTAIYYCTRQANWDFDVMDYWGQGTSVTVFAASGGGGSGGGGSGGGGSAGDIVMTQSQKFMSTSVGDRVSVTCKASQ
NVGTDVAWYQQKPGQSPKPLIYSASYRYRGVPDRFTGSGSGTDFTLTISNVQSEDLAEYFCQHYNSYPYTFGGGTKLEIKRAA
AAHHHHGAAEQKLISEEDLNGAA
>11
EVKLDESGGGLVQPGGSMKLSCVASGFTFSTYWMNWVRQSPEKGLEWVAEIRLKSNNYATHYAESVKGRFTISRDDSKSSVYL
QMNNLRAEDTGIYYCTREYYGSSPFDYWGRGTTLTVSSASGGGGSGGGGSGGGGSAGEIVLTQSPAIMSASPGEKVTMTCSAS
SSISYMHWYKQKPGTSPKRWIYDTSKLASGVPARFSSSGSGTDFTLRISRVEAEDVGVYYCAQNLELPWTFGGGTKLELKRAA
AAHHHHHHGAAEQKLISEEDLNGAA*
>23
EVQLEESGPDLVKPSQSLSLTCTVTGYSITSGYSWHWIRQFPGNKLEWMGYIHYRGSTNYNPSLKSRISITRDTSKNQFFLQL
NSVTTEDTATYYCGRGPGNWAWFAYWGQGTLVTVFSASGGGGSGGGGSGGGGSAGDIQMTQSPSSLSASLGDTITITCHASQD
IKSYLSWFQQKPGKSPKTLIYRANRLVDGVPSRFSGSGSGQDYSLTISNLDPEDIATYYCQQYSKFPWTFGGGTKVEIKRAAA
AHHHHHHGAAEQKLISEEDLNGAA
>35
EVKLLESGPGLVAPSQSLSITCTVSGFSLTSYGVHWVRQPPGKGLEWLGVIWAGGTPNYNSALMSRLSISKDNSKSQVFLKMN
SLQTDDTAMYYCAREPHGYYGYFDVWGAGTTVTVSAASGGGGSGGGGSGGGGSAGDIVLTQSPAIMSASPGEKVTMTCRASSS
VSSSYLHWYQQKSGASPKRWIYDTSKLASGVPARFSGSGSGTSYSLTISSMEAEDVATYYCQQGSSIPFTFGSGTKLEIKRAA
AAHHHHHHGAAEQKLISEEDLNGAA*
>47
EVQLVESGGDLVKPGGSLKLSCAASGFTFSDYYMYWVRQTPEKRLEWVATISDGGSYTYYPDSVKGRFTISRDNAKNNLYLQL
SSLKSEDTAMYYCARGGYYGNYLFPYWGQGTLVTVFAASGGGGSGGGGSGGGGSAGEIVLTQSPAIMSASLGEEITLTCSASS
SVSYMHWYQQKSGTSPKLLIYATSNLASGVPSRFSGSESGTFYSLTISSVEAEDAADYYCHQWSSYRTFGGGTKLEIKRAAAA
HHHHHHGAAEQKLISEEDLNGAA*
>59
EVQLEESGGGLVQPGGSLKLSCAASGFTFSSYGMSWVRQTPDKRLELVATINSNGGSTYYPASVKGRFTISRDNARNTLYLQM
SSLKSEDTAMYYCAREGKWAWLAYWGQGTLVTVFAASGGGGSGGGGSGGGGSAGENVLTQSPTTMAASPGEKITITCSASSSI
SYMHWYQQKPGTSPKRWIYDTSNLASGVPSRFSGSGSGTFYSLTISSVEAEDAADYYCHQWSSYPWTFGGGTKLAIKRAAAAH
HHHHHGAAEQKLISEEDLNGAA*
>71

EEKLEESGAELVRPGVSVKISCKGSGYTFTDYGIHWVKQTHAKSLEWIGVISTYYGDANYDQKFKGKATMTVDKSSSTAYMEL
ARLTSEDSAIYYCARSRYGSSYVDYWGQGTALTVSSASGGGGSGGGGSGGGGSAGEIVLTQSPAIMSASLGERVTMTCTANSS
VSSSYLHWYQQKPGSSPKLWIYSTSNLASGVPARFSGSGSGTSYSLTISSMEAEDAATYYCHQYHRSPWTFGGGTRLEIKRAA
AAHHHHHHGAAEQKLISEEDLNGAA*
>95
QIQLVQSGPELVKPGASVKVSCKASGYAFTGYNMYWVKQSHGRSLEWIGYIDPYNGGTSYNQKFKGKATLTVDKSSSTAYIHL
NSLTSEDSAVYFCARFRFDGYYAMDYWGQGTSVTVFSASGGGGSGGGGSGGGGSAGDIQMTQSPASLSASVGETVTITCRASE
NIYTYLAWYQQKQGKSPQLLVYNAKTLAEGVPLRFSGSGSDTQFSLKINSLQPEDFGSYYCQHHYATPPTFGGGTQLEIKRAA
AAHHHHHHGAAEQKLISEEDLNGAA
>12
EVQLVESGGGLVNLGGSLKLSCAASGFTFSSYYMSWVRQTPEKRLELVAAINSNGGSTYYPDTVKGRFTISRDNAKNTLYLQM
TSLKSEDTALYYCVRQWDSYYFDYWGQGTTLTVSSASGGGGSGGGGSGGGGSAGENVLTQSPAIMSASPGEKVTMTCSASSSV
TYMQWYQQKSGTSPKRWIYDTSKLASGVPARFSGSGSGTSYSLTISSMEAEDAATYYCQQWSSNPPTFGAGTKLDLKRAAAAH
HHHHHGAAEQKLISEEDLNGAA*
>24
EVQLQQSGAELVRPGTSLKMSCKTAGYTFTNYWINWVKQRPGHGLEWVGDIYPGGDYTNYNEQFKGKATLTADTSSSTAYMQL
NSLTSEDSAIYYCARRRDDYFDYWGQGTTLTVSSASGGGGSGGGGSGGGGSAGETTVTQSPASLSVATGEKVTIRCITSTDID
DDMSWYQQKPGEPPKLLISEGITLRPGVPSRFSSSGYGKDFVFTIENMLSEDVADYYCLQSDNLPLTFGAGTKLALKRAAAAH
HHHHHGAAEQKLISEEDLNGAA*
>56
EVQLLETGGGLVKPGGSLKLSCAASGFTFSSYAMSWVRQTPEKRLEWVATISSGGSHTYYPDSEKGRFTTSRDNAKNTLYLQM
SSLRSEDTAMYYCARQDYYGPYFDYWGQGTTLTVSAASGGGGSGGGGSGGGGSAGDIQMTQSPSSLSASLGGKVTITCKASQD
IKKYIAWYQHKPGKGPRLLIHYTSTLQPGIPSRFSGSGSGRDYSFSISNLEPEDVATYYCLQYDNLRTFGGGTKLELKRAAAA
HHHHHHGAAEQKLISEEDLNGAA*
>68
EVQLEESGPGLVQPSQSLSSTCTVSGFSLISYDVHWVRQSPGKGLEWLGVIWAGGSTNYNSALMSRLSISKDNSKSQVFLKMN
SLQTNDTAMYYCARDDYEAYWGQGTLVTVSSASGGGGSGGGGSGGGGSAGETTVTQSHKFMSTSVGDRVSITCKASQDVNTAV
AWFQQKPGHSPKLLISSASYRYTGVPDRFTGSGSGTDFTFTISSVQAEDLAVYYCQQHYSTPFTFGGGTKLEIKRAAAAHHHH
HHGAAEQKLISEEDLNGAA*
>80
QIQLKESGPGLVAPSQSLSITCTVSGFSLTAYGVNWVRQPPGKGLEWLGIIWAGGSTNYNSALMSRLSIRKDNSKSQVFLKMN
SLQTDDTAIYYCARDDSEAYWGAGTTVTVSSASGGGGFGGGGSGGGGSAGQIVLTQSPAIMSASPGEKVTISCSASSSVTFMY
WFQQKPGSSPKPWIYRTSNLASGVPPRFSGSGSGTSYSLTISSMEAEDAATYYCQQYHSYPPTFGGGTKLVLKRAAAAHHHHH
HGAAEQKLISEEDLNGAA*
>09
EVQLEESGTELARPGASVKMSCKASGYTFTSYTMHWVKQRPGQGLEWIGYINPNSGYTNYNQKFKDRATLTADKSSSTAYMQL
SSLTSEDSAVYYCARYGSEDWGQGTSVTVSSASGGGGSGGGGSGGGGSAGETTVTQSPASLSVATGEKVTIRCITSTDIDDDM
NWYQQKPGEPPKLLISEGNTLRPGVPSRFSSSGYGTDFVFTIENMLSEDVADYYCLQSDNMPYTFGGGTKLEIKRAAAAHHHH
HHGAAEQKLISEEDLNGAA*
>21
DVQLVESGGGLVKPGGSLKLSCAASGFTFSSYPMSWVRQTPEKRLELVAAINSNGGSTYYPDTVKGRFTISRDNAKNTLYLEM
SSLKSEDTALYYCARGTYGNYVQTYWGQGTLVTVSAASGGGGSGGGGSGGGGSAGDIQMTQSHKFMSTSVGERVSITCKASQD
VGTAVAWYQQKPKQSPKLLIYWASTRHTGVPDRFTGSGSGTDFTLTISNVQSEDLAVYYCQQHYSSPWTFGGGTELELKRAAA
AHHHHHGAAEQKLISEEDLNGAA*
>33
MITPSFGAFFLEIFNVKYLLPTAAAGLLLLAAHPAMAQVQLQQSGPEVVRPGVSVKISCKGSGYTFTDYAIHWVKQSHARSLE
WIGIISTYNGNTKYNQKFKGKATMTVDKSSSTAYMQLKSLTSEDSAVYYCARRGGDFHYAMDYWGQGTSVTVSSASGGGGSGG
GGSGGGGSAGEIVLTQSPAIMSASLGEEITLTCSASSSVSYMHWYQQKSGTSPKLLIYSTSNLASGVPSRFSGSGSGTFYSLT
ISSVEAEDAADYYCHQWSSYPWTFGGGTKLEIKRAAAAHHHHHHGAAEQKLISEEDLNGAA*
>45
EVKLVESGGDLVQPGGSRKLSCAASGFTFSDYVMAWVRQAPGKGPEWIAFISNLAYNIYYADTVTGRFTISRENAKNTLFLQM
TSLRSEDTAMYYCVRGGNHGGYFDYWGQGTTLTVSAASGGGGSGGGGSGGGGSAGDIVLTQSPSSLSASLGERVSLTCRASQD
IGSSLNWLQQEPDGTIKRLIYATSSLDSGVPKRFSGSRSGSDYSLTISSLESEDFVDYYCLQYASSPWTFGGGTKLDLKRAAA
AHHHHHHGAAEQKLISEEDLNGAA*
>57
EVQLQQSGAELVRPGVSVKISCKGSGYTFTDYGMHWVKQTHAKSLEWIGVISTYYGDANYNQKFEGKATMTVDKSSSTAYMEL
ARLTSEDSAIYYCARSRYGSSYVDYWGQGTTLTVSSASGGGGSGGGGSGGGGSAGETTVTQSPASLSVPTGEKVTIRCMTSTD
IDDDMNLYQQKPGEPPKLLISEGNTLRPGVPSRFSSSGYGTDFVFTIENMLSEDVADYYCLQSDNLPYTFGGGTKLAIKRAAA
AHHHHHHGAAEQKLISEEDLNGAA*
>69
EVQLQESGPGLVAPSQSLSITCTVSGFSLTDYGVHWVRQPPGKGLEWLGMIWGDGRTDYNSALKSRLSISKDNSKSQVFLKMN
SLQANDTAIYYCARKRGNYYAMDYWGQGTSVTVFAASGGGGSGGGGSGGGGSAGQIVLTQSPAIMSASPGEKVTMTCSASSSV
SYMYWYQQKPGSSPRLLIYDTSNLASGVPVRFSGSGSGTSYSFTISSMEAEDAATYYCQQWSSNPLTFGAGTKLELKRAAAAH
HHHHHGAAEQKLISEEDLNGAA*
>26
QIQLVQSGPELKKPGETVKISCKTSGYTFTNFGMNWVKQAPGKGLKWLGWINPYSGEPTYADDFKGRFAFSVETSASTAYLQI
NNLKNEDTATYFCARGTAAGYFDYWGQGTTLTVSSASGGGGSGGGGSGGGGSAGQIVLTQSPAIMSASPGEKVTMTCSASSSI

FYIHWYQQKSGTSPTSWIYDTSKLASGVPARFSGSGSGTSYSLTISTMEAEDAATYYCQQWSSDPPTFGSGTRLAIKRAAAAH
HHHHHGAAEQKLISEEDLNGAA*
>38
QVQMKESGPGLVAPSQSLSITCTVSGFSLTGYGVNWVRQPPGKGLEWLGMIWGDGSTDYNSALKSRLSISKDNSKSQVFLKMN
SLQTDDTARYYCAREYGNYFDYWGQGTTLTVSSASGGGGSGGGGSGGGGSAGEIVLTQSPALMSASPGEKVTMTCSASSSVSD
MYWYQQKPRSSPKPWIYLTSNLASGVPARFSGSRSGTSYSLTISSMEAEDAATYYCQQWSSNPLTFGAGTKLELKRAAAAHHH
HHHGAAEQKLISEEDLNGAA*
>50
EVQLEESGGGLVQPGGSLKLSCAASGFTFSSYGMSWVRQTPDKRLELVATINSNGGSTYYPDSVKGRFTISRDNAKNNLYLQM
SSLKSEDTAMYYCARDRGYGAMDYWGQGTSVTVSAASGGGGSGGGGSGGGGSAGDIVMTQAAPSVPVTPGESVSISCRSSESL
LHSNGNIYLYWFLQRPGQSPQLLIYRMSNLASGVPDRFSGSGSGTAFTLRISRVEAEDVGVYYCMQHLEYPYTFGGGTKLEIK
RAAAAHHHH
>62
QVQMKESGPGLVAPSQSLSITCTVSGFSLTGYGVNWVRQPPGKGLEWLGMIWGDGRTDYNSALKSRLSISKDNSKSQIFLKMN
SLQTDDTARYYCARDGYYDAMDYWGQGTSVTVSAASGGGGSGGGGSGGGGSAGDIVMTQSPSSLAMSVGQKVTMSCKSSQSLL
NNINQKNYLAWYQQKPGQSPKLLVYFTSTRESGVPDRFIGSGSGTDFTLTISSVQAEDLADYFCQQHYSTPLTFGAGTKLDLK
RAAAAHHHHHHGAAEQKLISEEDLNGAA*
>74
DVQLVESGGGLVQPGGSLKLSCAASGFIFSSYGMSWIRQTQDKRLELVATINSNGGRSYYPDSVKGRFTISRDNAKNTLYLQM
SSLKSEDTAMYYCARGGITTVVPFDYWGQGTTLTVFSASGGGGSGGGGSGGGGSAGQIVLTQSPALMSASPGEKVTMTCSASS
SVSYMFWYQQKPRSSPKIWIYLTSNLASGVPGRFSGSGSGTSYSLTISSMEAEDAATYYCQQWSSNPPTFGSGTKLELKRAAA
AHHHHHHGAAEQKLISEEDLNGAA*
>03
DVQLQESGPGLVKPSQSLSLTCTVTGYSITSDYAWNWIRQFPGNKLEWMGYISNSGITSYNPSLKSRISITRDTSRNQFFLQL
NSVTTEDTATYYCAPHWYAIDYWGQGTSVTVSSASGGGGSGGGGSGGGGSAGDIVMTQAAFSDPVTLGTSASISCRSSKSLLH
SNGITYLYWYLQKPGQSPQLLIYHMSNLASGVPDRFSSSGSGTDFTLRISRVEAEDVGVYYCAQNLELPYTFGGGTKLEIKRA
AAAHHHHHHGAAEQKLISEEDLNGAA*
>15
EVKLVESGGGLVKPGGSLKLSCAASGFTFSDYYMYWVRQTPEKRLEWVATITNGGSYTYYPDSVKGRFTISRDNAKNTLYLQM
SSLKSEDTAMYYCARHGGNYAMDYWGQGTSVTVSAASGGGGSGGGGSGGGGSAGDIVLTQTPKFLLVSAGDRVTITCKASQSV
SFDVAWYQQKPGQSPKLLIYYASNRYTGVPDRFTGSGYGTDFTFTISTVQAEDLAVYFCQQDYTSPLTFGAGTKLELKRAAAA
HHHHHGAAEQKLISEEDLNGAA*
>27
QVQMKESGPGLVAPSQSLSITCTVSGFSLTSYDISWIRQPPGKGLEWLGLIWTGGGTNYNSAFMSRLTISKDNSKSQVFLKMN
SLQTDDTAIYYCVRTSGLNYDDNYYSMDYWGQGTSVTVFAASGGGGSGGGGSGGGGSAGETTVTQSPASLSVAIGEKVTIRCI
TSTDIDDDMNWYQQKPGEPPKVLISEGNILRPGVPSRFSSSGYGTDFVFTIENMLSEDVADYYCLQSDNLPLTFGAGTKLEIK
RAAAAHHHHHHGAAEQKLISEEDLNGAA*
>39
DVQLVESGGDLVKPGGSLKLSCAASGFTFSTYGMSWVRQTPDKRLEWVATITNGGSYTYYPDSVKGRFTISRDNAKNTLYLQM
SSLKSEDTAIYYCARQYYVSSYDYAMDYWGQGTSVTVSAASGGGGSGGGGSGGGGSAGETTVTQSPASLSMAIGEKVTIRCIT
STDIDDDMNWYQQKPGEPPKLLISEGNTLRPGVPSRFSSSGYGTDFVFTIENMLSEDVADYYCLQSDNLPYTFGGGTKLELKR
AAAAHHHHHGAAEQKLISEEDLNGAA*
>36
EVQLLETGGDLVKPGGSLKLSCAASGFDFSSYDMSWVRQTPEKRLEWVAFISTTGGNIFYSETVKGRFTISRDNAKITLYLQM
SSLKSEDTAMYYCARQRVYDGYYRGHAMDYWGQGTSVTVSAASGGGGSGGGGSGGGGSAGDIVMTQSQKFMSTSVGDRVSVTC
KASQNVGTNVVWYQLKPGQSPNTLIYSASYRYTGVPDRFTGSGSGTDFTLTISNVQSEDLADYFCQQHYSTPWTFGGGTKLAL
KRAAAAHHHHHHGAAEQKLISEEDLNGAA*
>48
EVKLVESGGGLVQPGGSLKLSCAASGFTFSGYTMSWVRQTPERRLEWVATISNGGSYTYYPDSVKGRFTISRDNAKNNLYLQM
SSLRSEDTALYYCARHDPYNYDAVDYWGQGTSVTVSSASGGGGSGGGGSGGGGSAGEIVLTQSPVIMSASPGEKVTMTCSANS
SISYMHWYQQKPGTSPKRWIYDTSKLASGVPARFSGSGSGTSYSLTISSMEAEDAATYYCQQYSGYPLTFGAGTRLEIKRAAA
AHHHHHHGAAEQKLISEEDLNGAA*
>60
QVQLQQPGAELVRPGVSVKISCKGSGYTFTDYAMHWVKQSHAKSLEWIGIISTFNANTNYDQKFKGKATLTVDKSSSTAYMEL
ARLTSEDSAVYYCTRQLLRLAMDYWGQGTSVTVSAASGGGGSGGGGSGGGGSAGDVVMTQTPLTLSVTIGQPASISCKSSQSL
LDSDGKTYLNWLLQRPGQYPKRLIYLVSKLDSGVPDRFTGSGSGTDFTLKISRVEAEDLGVYYCWQGTHFPRTFGGGTKLEIK
RAAAAHHHHHGAAEQKLISEEDLNGAA*
>72
DVMLVESGGGLVKPGGSLKLSCAASGFTFSSYAMSWVRQTPEKRLEWVASISSGGTTYYPDSVKGRFTISRDNARNILYLQMS
SLRSEDTAMYYCARGGRYYGSSDDMDYWGQGTSVTVSAASGGGGSGGGGSGGGGSAGDIVMTQAAFSNPVTLGTSASISCRSS
KNLLHSNGITYLYWYLQRPGQSPQLLIYRVSNLASGVPNRFSGSESGTDFTLRISRVEAEDVGVYYCAQLLELPYTFGGGTKL
EIKRAAAAHHHHHHGAAEQKLISEEDLNGAA*
>84
QVQLQQSGPGLVKPSQSLSLTCSVTGFSITSGYYWTWIRQFPGNKLEWMGYISYDGSNNYNPSLKNRISITRDTSKNQFFLKL
NSVTTEDTATYYCANNYGNYHYAMEFWGQGTSVTVSSASGGGGSGGGGSGGGGSAGDIVLTQSPASLAVSLGQRATISCEASQ
SVDYDGGSYMIWYQQKPGQPPKLLIYAASNLESGIPARFSGSGSGTDFTLNIHPVEEEDAATYYCQQSIEDPPTFGGGTKLAI
KRAAAAHHHHHHGAAEQKLISEEDLNGAA*
>96

QVQLLETGGGLVKPGGSLKLSCAASGFTFSDYYMYWVRQTPEKRLEWVATISDGGSYTYYPDSVKGRFTISRDNAKNNLYLQM
SSLKSEDTAMYYCAREGENRYGGMDYWGQGTSVTVSSASGGGGSGGGGSGGGGSAGDVVMTQTPKFLLVSAGDRVTITCKASQ
SVSNDVAWYQQKAGQSPKMLIYYASNRYTGVPDRFTGSGYGTDFTFTISTVQAEDLAVYFCQQDYRTPLTFGAGTKLELKRAA
AAHHHHHHGAAEQKLISEEDLNGAA*
>06
EVKLLESGPGLVAPSQSLSITCTVSGFSLTGYGVNWIRQSPGKGLEWLGMIWGDGGTDYNSGLKSRLSISKDNSKSQVFLKMN
SLQTDDTAMYYCARERGDYYGSSYVDYWGQGTTLTVSAASGGGGSGGGGSGGGGSAGQIVLTQSPAFMSASLGEEITLTCSAS
SSVVYMHWYQQKSGTSPKLLIYSTSNLASGVPSRFSGSGSGTFYSLTISSVEAEDAADYYCHQWSSYPLTFGAGTKLELKRAA
AAHHHHHHGAAEQKLISEEDLNGAA*
>18
QVQLQQSGAELMKPGASVKISCKASGYRFSSYWIEWVKQRPGHGLEWIGEILPGSGSTNYNEKFEGKATLTVDKSSSTAYMEL
SSLTSEDSAVYYCARSGYGSSYVDYWGQGTTLTVSSASGGGGSGGGGSGGGGSAGDIVMTQSQKFMSTSVGDRVSVTCKASQN
VGTNVAWYQQKPGQSPKALIYSASYRYSGVPDRFTGSGSGTDFTLTISNVQSEDLAEYFCQQYNSYPLTFYAGTKLELKRAAA
AHHHHHHGAAEQKLISEEDLNGAA*
>30
EVMLVESGGDLVKPGGSLKLSCAASGFTFSRYAMSWVRQTPDKRLEWVATISSGGGYTYYLDSVEGRFTISRDNAKNTLYLQM
SSLKSEDTAMYYCARQEGLGRAMDYWGQGTSVTVFAASGGGGSGGGGSGGGGSAGEIVLTQSPAIMSASPGEKVTMTCSASSS
VSYMHWYQQKSGTSPKRWIYDTSKMTSGVPARFSGSGSGTSYSLTISRMEAEDAATYYCQQRSFYPRTFGGGTKLEIKRAAAA
HHHHHHGAAEQKLISEEDLNGAA*
>42
QVQLQQSGAELVKPGASVKLSCKASGYTFTNYYMYWVKKRPGQGLEWIGEINPTIGGTKFNEKFMSKAILTVDKSSSTAYMQL
SSLTSEDSAVYYCTISPYGNYVFDYWGQGTTLTVFAASGGGGSGGGGSGGGGSAGENVLTQSPAIMSASLGERVTMTCSASSS
VSYMHWYQQKPGTSPKRWIYDTSKLASGVPARFSGSGSGTSYSLTISSMGAEDAADYYCHQWSSYPCTFGAGTKLAIKRAAAA
HHHHHHGAAEQKLISEEDLNGAA*
>54
QVQLQQSGPELVKPGASVKMSCKASGYTLSSYVMYWVKQKPGQGLELIGYINLYNDGTKYNEKFKGKATLTSDKSSSTAYMEL
SSLTSEDSAVYYCARWGSYYGNYFDYWGQGTTLTVSSASGGGGSGGGGSGGGGSAGDIVMTQSQKFMSTSVGDRVSVTCKASQ
HVGTNVAWYQQKPGQSPKALIYSASYRYSGVPDRFTGSGSGTDFTLTISNVQSEDLAEYFCQQYNIYPLTFGGGTKLAIKRAA
AAHHHHHHGAAEQKLISEEDLNGAA*
>78
EVQLLETGGGLVKPGGSLKLSCAASGFTFSSYAMSWVRQSPEKRLEWVAEISSGGSYTYYPDSVKGRFTISRDNAKNTLYLQM
SSLKSEDTAMYYCARHGGNYAMDYWGQGTSVTVFAASGGGGSGGGGSGGGGSAGDIVLTQSPASLAVSLGQRATISCKASQSV
DYDGDSYMNWYRQKPGQPPKLLIYAASNLESGIPARFSGSGSGTDFTLNIHPVEEEDAATYYCQQSNEDPWTFGGGTKLEIKR
AAAAHHHHHHGAAEQKLISEEDLNGAA*
>07
EVKLVESGGGLVKPGGSLKLSCAASGFDFSSYDMSWVRQTPEKRLEWVAFISTGGGNTFYPDTVKGRFTISRDNAKITLYLQM
SSLKSEDTAMYYCARQRVYDGYYRGHAMDYLGQGTSVTVFSASGGGGSGGGGSGGGGSAGDIVMTQSPASLAVSLGQRATISC
KASQSVDYDGDSYMNWYQQKPGQPPKLLIFAASNLESGIPARFSGSGSGTDFTLNIHPVEEEDAATYYCQQSNEYPYTFGGGT
RLDIKRAAAAHHHHHHGAAEQKLISEEDLNGAA*
>19
EVQLLETGGGLVKPGGSLKLSCAASGFTFSDYYMYWVRQTPEKRLEWVATISDGGYYTYYPDSVKGRFTISRDNAKNNLYLQM
SSLRSEDTAIYYCARGFTNYFDNWGQGTTLTVSAASGGGGSGGGGSGGGGSAGDIVMTQSQKFMSTSVGDRVSVTCKASQNVG
TNVAWYQQKPGQSPQALIYSASYRYSGVPDRFTGSGSGTDFTLTISNVQSEDLAEYFCQQYNSYPLATFGAGTKLELKRAAAA
HHHHHHGAAEQKLISEEDLNGAA*
>51
QVQLQQPGAELVRSGASVKLSCTASGFNIKDYYMHWVKQRPEQGLEWIGWIDPENGDTEYAPKFQGKATMTADTSSNTAYLQL
SSLTSEDTAVYYCNYYGSSYAMDYWGQGTSVTVFAASGGGGSGGGGSGGGGSAGEIVLTQSPAIMSASPGEKVTISCSASSSV
SYMYWYQQKPGSSPKPWIYSTSYLASGVPARFSGSGSGTSYSLTISSMEAEDAATYYCQQYHSYPTFGGGTKLEIKRAAAAHH
HHHHGAAEQKLISEEDLNGAA*
>63
EVQLQQSGAELVKPGTSVKLSCKASGYTFTNYWMHWVKQRPGQGLEWIGEINPNNGRTFYNEKFKTKATLTVDTSSSTAYLQL
SRLTSEDSAVYFCTRGRYYGMDYWGQGTSVTVSSASGGGGSGGGGSGGGGSAGETTVTQSPASLSVATGEKVTIRCITNTEID
DDMNWYQQKPGEPPKLLISEGNILRPGVPSRFSSSGYGTDFVFTIENMLSEDVADYYCLQSDNLPWTFGGGTKLEIKRAAAAH
HHHHHGAAEQKLISEEDLNGAA*
>75
QVQLQQPGPEVVRPGVSVKISCKGSGYRFTDYAMHWVRQSHAKSLEWIGFISTYYGDGTYNQKFKGKATMTVDKSSSTAYMEL
ARLTSEDSAIYYCARSGYGSSYVDYWGQGTTLTVFAASGGGGSGGGGSGGGGSAGENVLTQSPAIMAASLGQKVTMTCSANSS
VSSIFLHWYQQKSGASPQPLIHRTSNLASGVPPRFSGSGSGTSYSLTISSVEAEDDATYYCQQWSGYPYTFGGGTKLELKRAA
AAHHHHHHGAAEQKLISEEDLNGAA*
>04
DVQLVESGGGLVKPGGSLKLSCAASGFTFSSYYMSWVRQTPEKRLELVAAINSNGGSTYYADTVKGRFTIYRDNAKNTLYLQM
NSLKSEDTAMYYCASLLLRDNYFDYWGQGTTLTVSSASGGGGSGGGGSGGGGSAGEIQMTQTTSSLSASLGGKVTISCKPGIP
SRFSGSGSGRDYSFSISNLAPEDIATYYCLQYDNLHTFGGGTKLEIKRAAAAHHHHHHGAAEQKLISEEDLNGAA*
>16
QVQLKQSGAEVVRPGVSVKISCKGSGYTFTDYPMHWVKQSHAKSLEWIGFISTYYGDTNYNQKFKGKATMTVDKSSSTAYMEL
VRLTSEDSAIYYCARSGYGSSYVDYWGQGTTLTVSAASGGGGSGGGGSGGGGSAGETTVTQSPASLSVATGEKVTIRCITSTD
IDDDMNWYQQKPGEPPKLLISEGNTLRPGVPSRFSSSGYGTDFVFSIENTLSEDVADYYCLQSDNMPLTFGAGTKLELKRAAA
AHHHHHHGAAEQKLISEEDLNGAA*

>40
EVKLMESGGGLVQPGGSRKLSCAASGFTFSSFGMHWVRQAPDKGLEWVAYISSGSSIIYYADTVKGRFTISRDNPKNTLFLQM
TSLRSEDTAMYYCARGNYYFDYWGQGTTLTVSAASGGGGSGGGGSGGGGSAGEILLTQSPALMSASPGEKVTMTCSASSSVTY
MQWYQQKSGTSPKRWIYDTSKLASGVPARFSGSGSGTSYSLTISSMEAEDAATYYCQQWSSNPPTFGAGTKLELKRAAAAHHH
HHHGAAEQKLISEEDLNGAA*
>52
EVKLLESGPGLVKPSQSLSLTCTVTGYSITSDYAWNWIRQFPGNKLEWMGYINYSGGTSYNPSLKSRISITRDTSKNQFFLQL
NSVTIEDTATYYCAISGGNYVLYWYFHVWGAGTTVTVFAASGGGGSGGGGSGGGGSAGDIVLTQSQKFMSTSVGDRVTVTCKA
SQNVGTNVAWYQQKPGQSPKALIYSASYQNNGVPDRFTGSKSGTDFTLSISNVQSEDLAEYFCQQYHSYPYTFGGGTMLEIKR
AAAAHHHHHHGAAEQKLISEEDLNGAA*
>64
QVQLQQSGAELMKPGASVKISCKATGYTFSSYWIEWVKQRPGHGLEWIGEILPGSDSTNYNEKFKGKATFTADTSSNTAYMQL
SSLTSADSAVYYCARGGALYGSSLDYAMDYWGQGTSVTVSSASGGGGSGGGGSGGGGSAGETTVTQSPASLSVATGEKVTIRC
ITSTDIDDDMNWYQQKPGEPPKLLISEGNTLRPGVPSRFSSSGYGTDFVFTIANMLSEDVADYYCLQSDNLPYTFGGGTKLVL
KRAAAAHHHHHHGAAEQKLISEEDLNGAA*
>01
EVKLMESGGGLVQPGGSRKLSCAASGFTFSDYGMAWVRQAPGKGPEWVAFISNLAYSIYYADTVTGRFTISRENAKNTLYLEM
SSLRSEDTAMYYCARHGGNYAMDYWGQGTSVTVFAASGGGGSGGGGSGGGGSAGDIVLTQSPASLAVSLGQRATISCRASQSV
STSSYSYMHWYQQKPGQPPKLLIYAASNVESGVPARFGGSGSGTDFSLTIHPVEEDDIAMYFCQQTRKVPWTFGGGTKLVIKR
AAAAHHHHHHGAAEQKLISEEDLNGAA*
>13
DVQLQESGPGLVAPSQSLSITCTVSGFSLTDYGVNWVRQSPGRGLEWLGMIWGDGTTDYNSALKSRLSISKDNSKSQLFLKMN
SLQTDDTARYYCAKTGRENYALDYWGQGTSVTVFAASGGGGSGGGGSGGGGSAGETTVTQSPASLSVATGEKVTIRCITSTDI
DDDMNWYQQKPGEPPKLLISEGNTLRPGVPSRFSSSGYGTDFIFTIENTLSEDVADFYCLQSDNVPLTFGGGTKLELKRAAAA
HHHHHHGAAEQKLISEEDLNGAA*
>25
EVKLDESGGGLVKLGGSLKLSCAASGFTFSSYYMSWVRQTPEKRLELVAAINSNGGSTYYPDTVKGRFTISRDNAKNTLYLQM
SSLKSEDTALYYCARQWGSYYFDYWGQGTTLTVFSASGGGGSGGGGSGGGGSAGEIVLTQSPALMSASPGEKVTMTCSTSSSV
SYMYWYQQKPRSSPKPWIYLTSNLASGVPARFSGSGSGTSYSLTISSMEAEDAATYYCQQWSRNPLTFGAGTKLELKRAAAAH
HHHHHGAAEQKLISEEDLNGAA*
>37
EVKLVESGGGLVQPGGSRKLSCAASGFTFSSSGLHWVRQAPEKGLEWVAYISGGSNTIYYADTLKGRFTISRDNPKNTLFLQM
TSLRSEDTAMYYCARKGLRLRDYAVDYWGQGTSVTVSAASGGGGSGGGGSGGGGSAGETTVTQSPSSMYASLGERVTITCKAS
QDIKTYLTWYQQKPWKSPKTLIYSATNLADGVPSRFSGSGSGQDYSLTISSLESDDTATYYCLQHVESPWTFGGGTKLEIKRA
AAAHHHHHHGAAEQKLISEEDLNGAA*
>49
EVQLQESGPGLVKPSQSLSLTCTVTGYSITSDLAWNWIRQFPGNKLEWMGYITYSGSTNYNPSLKSRMSLIRDTSKNQFFLQL
NSVTTEDTATYYCASLQFGLHGYGMDYWGQGTSVTVSSASGGGGSGGGGSGGGGSAGDVVMTQTPLSLPVSLGDQASISCRSS
QSLVHSNGNTYLHWYLQKPGQSPKLLIYKVSNRFSGVPDRFSGSGSGTDFTLKISRVEAEDLGVYFCSQSTHVPLTFGAGTKL
ELKRAAAAHHHHHHGAAEQKLISEEDLNGAA*
>61
QVQLKESGAELMKPGASVKISCKATGYTFSSYWIEWVKQRPGHGLEWIGEILPGSGNTNYTEKFKGKATFTADSSSNTVYMQL
SSLTSEDSAVYYCARTARASYAMDYWGQGTSVTVSSASGGGGSGGGGSGGGGSAGETTVTQSPASLSVATGEKVTIRCITSTD
VDDDMSWYQQKPGEPPKFLISEGNTLRPGVPSRFSSSGYGTDFVFTIENTLSEDVADYYCLQSDNMPYTFGGGTKLAIKRAAA
AHHHHHGAAEQKLISEEDLNGAA*
>73
QVQLKQSGPGLVKPSQSLSLTCTVTGYSISSDYAWNWIRQFPGNKLEWMGYIAYSDTTYYNPSLKSRISITRDTSKNQFFLQL
NSVTTEDTATYYCARFPLTSYVYYYTMDYWGQGSSVTVSSASGGGGSGGGGSGGGGSAGETTVTQSPASLSMAIGEKVTIRCI
TSTDIDDDMNWYQQKPGEPPKLLISEGNTLRPGVPSRFSSSGYGTDFVFTIENMLSEDVADYYCLQSDNLPYTFGGGTKLELK
RAAAAHHHHHHGAAEQKLISEEDLNGAA*
>02
QVQLKESGPGLVAPSQSLSITCTVSGFSLTGYGVNWVRQPPGKGLEWLGMIWGDGSTDYNSALKSRLSISKDNSKSQVFLKMN
SLQTDDTARYYCATSGYSYAMDYWGQGTSVTVFSASGGGGSGGGGSGGGGSAGDIQMTQSPSSMSASLGDRITITCQATQDIV
KNLNWYQQKPGKPPSFLIYYATELAEGVPSRFSGSGSGSDYSLTISNLESEDFADYYCLQFYDFPYTFGGGTKLELKRAAAAH
HHHHHGAAEQKLISEEDLNGAA*
>14
FGAFFLEIFNVKYLLPTAAAGLLLLAAQPAMAEVKLEESGGGLVQPGGSLKLSCATSGFTFSDYYMYWVRQTPEKRLEWVAYI
SSADNTYYPDSVKGRFTISRDNARNILYLQMSSLRSEDTAMYYCVRGYDYFDYWGQGTTLTVSSASGGGGSGGGGSGGGGSAG
DIQMTQSPSSLSASLGERVSLTCRASQEISGYLSWLQQKPDGTIKRLIYAASTLDSGVPKRFSGSRSGSDYSLTISSLESEDF
ADYYCLQYASYPLTFGAGTKLELKRAAAAHHHHHHGAAEQKLISEEDLNGAA*

>85
EVQMKQSGPGLVAPSQSLSITCTVSGFSLSRYTVHWVRQPPGKGLEWLGMIWGGGSTDYNSALKSRLTISKDNSKSQVFLKMN
SLQTDDTAMYYCARRGNYWHFDVWGAGTTVTVSAASGGGGSGGGGSGGGGSAGDIVLTQSPASLAVSLGQRATISCRASESVD
NYGISFMNWFQQKPGQPPKLLIYAASNQGSGVPARFSGSGSGTDFSLTIDPVEADDAATYYCQQNNEDPYTFGGGTKLEIKRA
AAAHHHHHHGAAEQKLISEEDLNGAA*
>86

EVKLMESGGGLVKPGGSLKLSCAASGFTFSSYAMSWVRQTPEKRLEWVASISSGGSTYYPDSVKGRFTISRDNARNILYLQMS
SLRSEDTAMYYCARGDYGGYAMDYWGQGTSVTVSAASGGGGSGGGGSGGGGSAGENVLTQSPAIMSASPGEKVTMTCSATSSV
SYMHWFQQTSSTSPKLWIYDTSKLTSGVPGRFSGSGSGNSYSLTISSMEAEDVATYYCFQGSGYPLTFGTGTKLELKRAAAAH
HHHHHGAAEQKLISEEDLNGAA*
>87
EVQLQQSGAELAKPGASVKMSCKASGYTFTTYWMHWLKQRPGQGLEWIGYINPSTGYTEYNQNFKDKATLTADKSSSTAYMQL
SSLTSEDSAVYYCARSYYGSSYFDYWGQGTTLTVSSASGGGGSGGGGSGGGGSAGQIVLTQSPALMSASPGEKVTMTCSASSS
VTYMYWYQQKPRSSPKPWIYLTSNLASGVPVRFSGSGSGTSYSLTISRMEAEDAATYYCQQWSSYTWTFGGGTKLAIKRAAAA
HHHHHHGAAEQKLISEEDLNGAA
>88
DVQLVESGGGLVQPGGSRKLSCAASGFTFSSFGMHWVRQAPEKGLEWVAYISSGSSTIYYADTVKGRFTISRDNPKNTLFLQM
TSLRSEDTAMYYCARRGFYYHGSSYYYAMDYWGQGTSVTVSSASGGGGSGGGGSGGGGSAGEIVLTQSPAIMSASPGEKVTMT
CSASSSVSYMQWYQQKSGTSPKRWIYDTSKLASGVPARFSGSGSGTSYSLTISSMEAEDAATYYCQQWSSNPPTFGAGTKLEL
KRAAAAHHHHHHGAAEQKLISEEDLNGAA*
>89
QIQLVQSGAELVRPGVSVKISCKGSGYTFTDYGVHWIKQTHAQSLEWIGVISTYYGDANYNQKFRGKATLTVDKSSSTAYIYL
ARLTSEDSAIYYCARSRYGSNYVDHWGQGTTLTVSAASGGGGSGGGGSGGGGSAGENVLTQSPAITAASLGQKVTLTCSASSS
VSYMHWFQQKSGTSPKRWIYDTSKLASGVPGRFSGSGSGTSYSLTIGTLEAEDVATYYCQQGSSLPYTFGGGTKLELKRAAAA
HHHHHHGAAEQKLISEEDLNGAA*
>91
EVQLLETGGGLVKPGGSLKLSCAASGFTFSSYGMSWVRQTPDKRLEWVATITNGGRYTYYPDSVKGRFTISRDNAKNTLYLQM
SSLKSEDTAMYYCARHYYGSTYDYAMDYWGQGTSVTVSAASGGGGSGGGGSGGGGSAGDIVMTQAAFSNPVTLGTSASISCRS
SKSLLHSHGITYLYWYLQKPGQSPQLLIYQMSNLASGVPDRFSSSGSGTDFTLRISRVEAEDVGVYYCAQNLELPYTFGGGTK
LEIKRAAAAHHHHGAAEQKLISEXDLNGAA
>92
EVQLVESGGGLVQPGGSLKLSCAASGFTFSTYGMSWVRQTPDKRLELVATINSNGGSTYYPDSMKGRFTMSRDNAKNTLYLQM
SSLKSEDTAMYYCARESSYSWFAYWGQGTLVTVFSASGGGGSGGGGSGGGGSAGDVVMTQTPKFLLVSAGDRVTITCKASQSV
NNDVAWYQQKPGQSPKLLIYYASTRYSGVPDRFTGSGYGTDFTFTISTVQAEDLAVYFCQQDYSSPYTFGGGTKLEIKRAAAA
HHHHHHGAAEQKLISEEDLNGAA*
>93
DVQLQESGPGLVAPSQSLSITCTVSGFSLTSYGVHWVRQPPGKGLEWLGVIWAGGSTNYNSALMSRLSIIRDISKSQVFLKMN
SLQTDDTSMYYCARDDYEAYWGQGTLVTVSAASGGGGSGGGGSGGGGSAGDIQMTQAAFSNPVTLGTSASISCRSSKSLLHSN
GITYLYWYLQRPGQSPQILIYRMSNLASGVPDRFSGSGSGTAFTLRISRVEAEDVGVYFCMQHLENPYTFGGGTKLEIKRAAA
AHHHHHHGAAEQKLISEEDLNGAA*

**Samples type**	Final library
**Number of positive clones**	96
**Number of non-coding clones**	8
**Number of exploitable sequences**	88
**Number of different sequences**	88
**Number of identical sequences**	0
**Number of different CDR3 clones**	76 VH – 77 VK
**Final result**	85% CDR3 diversity; 100% Total Diversity
