# Supplementary figures and images for: Development of Anti-LRRC15 Small Fragments for Imaging Purposes Using a Phage-Display ScFv Approach
Source: Int J Mol Sci. 2022 Oct 21;23(20):12677. doi: 10.3390/ijms232012677 (PMC9604383; doi:10.3390/ijms232012677)

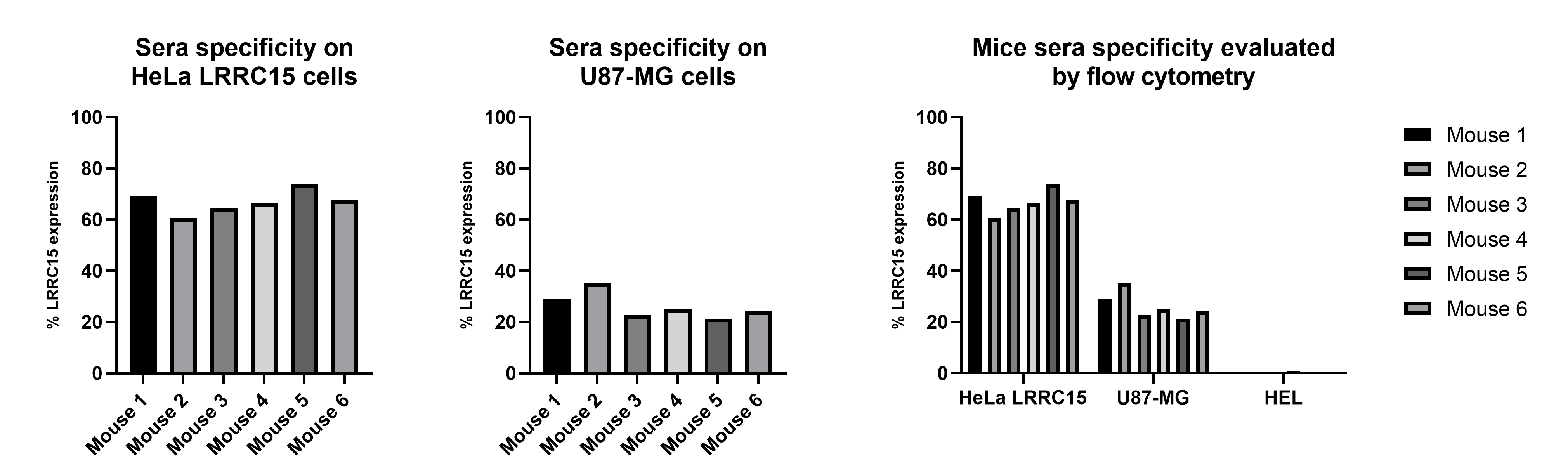

Supplement: Supplementary file 1 [file ijms-23-12677-s001.zip › ijms-1927729-supplementary/SM/FigureS1.png]

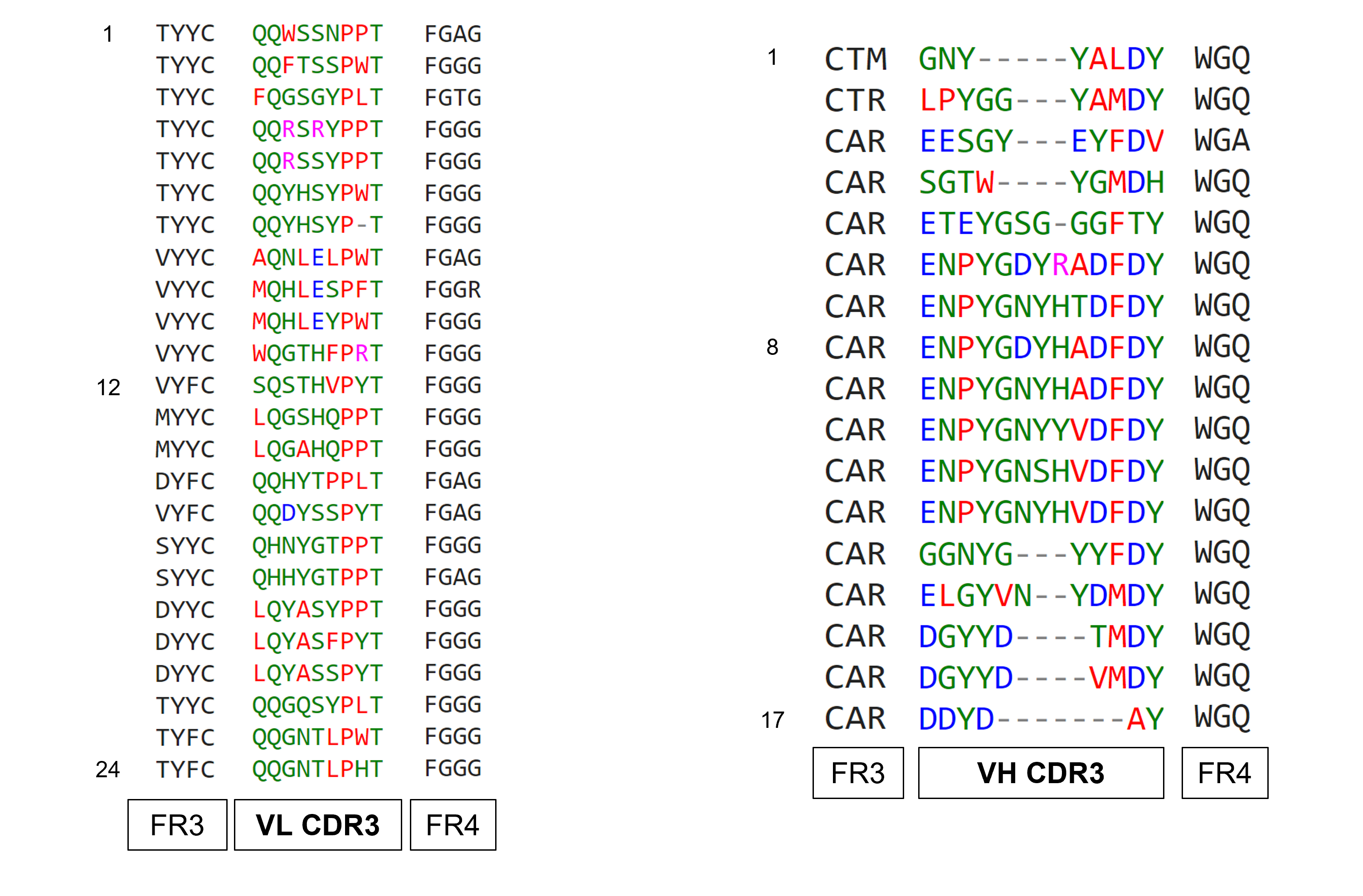

Supplement: Supplementary file 1 [file ijms-23-12677-s001.zip › ijms-1927729-supplementary/SM/FigureS2.png]
